# Relationship Between Breastfeeding Duration and Exclusivity on Various Language Milestones in United States Children Aged 3–5 Years

**DOI:** 10.3390/children12060719

**Published:** 2025-05-30

**Authors:** Malika Goel, Sowmya Jayachandran, Sandhya J. Kadam, Rhythm Sharma

**Affiliations:** 1 Department of Pediatrics, University of California, San Franciso, CA 94143, USA; 2 Deepam Medfirst Hospital, Chennai 600100, India; sowmyajaya20@gmail.com; 3 Family HealthCare Network, Visalia, CA 93291, USA; hellosandhya7@gmail.com; 4 Corewell Health at William Beaumont University Hospital, Royal Oak, MI 48073, USA; sharmarhythm65@gmail.com

**Keywords:** language milestones, breastfeeding, exclusive breastfeeding, school readiness, preschool age group

## Abstract

**Background/Objectives:** Breastfeeding has been positively associated with development of various developmental and cognitive outcomes. Although not fully understood, psychosocial, environmental, nutrients (docosahexaenoic acid) etc., have been proposed as reasons. There is a paucity of studies that have looked at individual language milestones and language milestones associated with school readiness in the age group of 3–5 years old in a nationally representative sample. This study aimed to analyze the language milestones association with breastfeeding in this group of children. **Methods:** The dataset was obtained from the National Survey of Child Health (NSCH) 2022–2023 combined sample. Overall, 22,866 children met the inclusion criteria. Secondary analysis of the NCSCH data was done using multinomial regression models amongst four breastfeeding categories (never breastfed, breastfed less than 6 months, breastfed until 6 months but not exclusively, exclusive breastfeeding) with 16 language variables. **Results:** The results of the study show that children in the breastfeeding categories (breastfed until 6 months but not exclusively and exclusive breastfeeding until 6 months) had a positive association across many language variables including variables used to analyze school readiness. Individual language variables’ adjusted odds ratio and significance has been analyzed. The reference category was never breastfed. **Conclusions:** The study results support positive association of breastfeeding with language variables. Per our review, limited research has been reported where so many individual language variables have been analyzed in a nationally representative sample across four different breastfeeding categories.

## 1. Introduction

The development of language in a child is influenced by both genetic and environmental factors [1]. Among the environmental factors, the role of breastfeeding has been studied as a potentially modifiable early-life exposure that influences neurodevelopmental and cognitive outcomes including the development of language skills.

Evidence suggests that both duration and exclusivity of breastfeeding may be important in language development. In 2007, Dee et al. [2] analyzed data from over 22,339 children aged 10 to 71 months using the 2003 National Survey of Child Health (NSCH) data. The study found that children who were breastfed for >3 months had significantly fewer concerns related to development of expressive and receptive language, fine and gross motor skills compared to children who were never breastfed. This study was based on four outcome parent reported variables on questions about concern for development in each of the above domain. However, this study did not have data on whether exclusive breastfeeding was present or not [2].

Similarly, another study done in a UK based cohort by Quigley et al. established that breastfeeding is associated with an improved cognitive outcome at 5 years of age [3].

A dose–response relationship was observed between the duration of exclusive breastfeeding and cognitive outcomes in a study conducted by Wiesław Jędrychowski et al. in 2011 [4]. Children who were exclusively breastfed for up to three months, 4–6 months and more than 6 months had IQ scores averaging 2.1 points (95% CI: 0.24–3.9), 2.6-point increase in IQ (95% CI: 0.87–4.27) and 3.8 points (95% CI: 2.11–5.45) higher than their non-exclusively breastfed counterparts [4]. In 2013, Mandy B. Belfort et al. also found that longer duration of exclusive breastfeeding is associated with better receptive language at age 3 and with higher verbal and nonverbal IQ at age 7 [5].

However, an analysis by Jonsdottir et al. found no sustained benefit of longer duration of exclusive breastfeeding on receptive and expressive language at 30–35 months of age [6].

The mechanism behind better developmental outcomes and breastfeeding is not fully understood. The psychosocial and environmental factors related to breastfeeding, such as mother–child interaction and bonding may play a part. Innis et al. found that that docosahexaenoic acid (DHA), present in breast milk could also influence the development of central nervous system which could be associated with better developmental outcomes including language [7].

Despite these findings, there remains a paucity of studies that examine the impact of breastfeeding across multiple, specific language domains during the preschool years. This is particularly the case when using nuanced breastfeeding categories that distinguish between exclusivity and duration, having a nationally representative sample and a large sample size. Furthermore, there is paucity of studies that have examined outcomes such as phonemic awareness, verbal memory, and emotional vocabulary—skills that are increasingly recognized as foundational for academic readiness during the preschool years [8].

The current study’s objective is to analyze the relationship between four breastfeeding exposures (never breastfed, breastfed <6 months, breastfed to 6 months but not exclusively, and exclusively breastfed to at least 6 months) and 15 language-related outcomes in children aged 3–5 years. The study plans to use data from the 2022–2023 National Survey of Children’s Health (NSCH), a large, nationally representative sample of Unites States (U.S.) children. The language outcomes include foundational skills (e.g., spoken words, comprehension), as well as higher-order language abilities (e.g., rhyming, phonemic awareness, storytelling, and emotional vocabulary). By doing so, this study offers a more nuanced and detailed understanding of how early feeding practices relate to language development during a critical period of early childhood.

## 2. Materials and Methods

### 2.1. Objectives

The study aimed to analyze the relationship between breastfeeding between 0–6 months of age and language development in children between 3–5 years of age.

### 2.2. Study Sample

The study sample was taken from the National Survey of Child Health (NSCH) 2022–2023 combined dataset [9]. The survey was conducted from July 2022 to January 2023 for the NSCH 2022 and from June 2023 to Jan 2024 for the NSCH 2023. The survey data have been adjusted and weighting provided for non-institutionalized children across various states between 0–17 years of age. A web-based or mail-based survey questionnaire was mailed to various U.S. households and data were collected for a randomly selected single child belonging to that household. The questionnaire is based on multiple health aspects of a child life including demographics, family dynamics, health parameters and factors thought to influence those health parameters, etc. [10].

The weighted response for 2023 NSCH is 35.8% and for 2022 NSCH is 39.1%. A total of 109,265 surveys were completed by caregivers of children for the 2022-2023 combined NSCH data. The detailed methodology can be found in the NSCH methodology reports [11,12]. Our study included children between 3 to 5 years for whom information of breastfeeding was available. A total of 22,876 children between the age of 3–5 years had information available on breastfeeding in the combined 2022–2023 NSCH data.

The authors have analyzed these data for research purposes and did not make any attempts or accidentally identify any person, and complied with the data use agreement as stated on the NSCH website. The dataset is a national dataset which is available publicly, the authors did not take individual institution’s IRB approval.

The Census Bureau and Health Resources and Services Administration’s Maternal and Child Health Bureau (HRSA MCHB) take extraordinary measures to assure that the identity of survey subjects cannot be disclosed. Before releasing any statistics to the public, the Census Bureau reviews them to make sure none of the information or characteristics could identify someone. Before releasing any statistics to the public, the Census Bureau reviews them to make sure none of the information or characteristics could identify someone.

### 2.3. Study Design and Description of Variables

This was a retrospective cohort analysis based on secondary analysis of National Survey of Child Health (NSCH) combined 2022–2023 survey. The survey collected data about exclusivity and duration breastfeeding between 0–6 months of age from children who were between 0–5 years at the time of survey.

The exposure variable was breastfeeding at 0–6 months, categorized into four possible responses namely, exclusive breastfeeding for 6 months, breastfeeding done until 6 months but not exclusively, breastfed less than 6 months, and never breastfed. The main covariates were the sex and race of child.

This was compared with development of 15 different language milestones between 3 to 5 years of age. The language variables are the outcome variables in this study. These data were collected during the survey across both receptive and expressive skills. The outcome variables also included language variables associated with school readiness in the 3–5 year age group.

The language variables collected either had a binary response (yes, no) or were graded on a Likert scale. The responses were given by primary caregiver of the child.

For example, language variables including use of two words together, use of three words together, asks questions: Why and How, asks questions: Who, What, When, Where, able to tell a story, understand ‘no’, follow verbal commands, point to things, follow two step commands, understand ‘in’, ‘on’, and ‘under’ had binary response of yes or no.

The variable ‘rhyme words’ had four possible responses: able to rhyme very well, able to rhyme somewhat well, not able to rhyme well, does not rhyme.

The rest of the language variables, namely, child can come up with words starting with the same sound, child can recognize beginning sound of a word, child can recognize and name their own emotions, and child can explain things child has seen or done, had five possible responses: always, most of the time, about half of the time, less than half of the time, and never.

### 2.4. Statistical Analysis

The analysis was done using IBM SPSS statistical software, version 29.0.2.0 [13]. The exposure variable is the duration and exclusivity of breastfeeding and outcome variables are the 15 language variable described above. Weighted analysis was done and adjusted for race and sex.

Descriptive statistics were used for providing distribution of different variables and comparing the number of each outcome variable and covariate across different breastfeeding categories. When using outcome or language variables with binary outcomes, binary logistic regression was performed to analyze and report the adjusted odds ratio between breastfeeding categories and language variables. Missing values were handled by listwise deletion, as all missing data were less than five percent within the large sample size. Similarly, with language variables having multiple possible responses, multinomial regression was done. Confidence intervals (CIs) and *p*-values have been reported with each analysis. A result was considered to be statistically significant when the *p*-value was less than 0.05 or the CI was greater than 95%.

## 3. Results

### 3.1. Demographics

The total sample size included in the study was 22,866 in the age group of 3–5 years. Weighted analysis was used during the study. Table 1 shows the distribution of children across four independent variables. Out of 22,866 children, 33% children were 3 years old, 33.8% children were 4 years old, and 33.1% children were 5 years old at the time of survey. Males were 51.2%. Amongst the four breastfeeding categories, 19.8% never received breast milk, 23% were fed breast milk but less than 6 months, 28.3% received breast milk up to 6 months but not exclusively, and the rest (28.9%) were exclusively breastfed. A total of 69.4% of the children were white. The rest of the racial distribution can be seen in Table 1.

As mentioned above, the breastfeeding categories were compared with 15 language variables. Table 2 shows the distribution of language milestones and age, sex, and race against the four breastfeeding categories.

### 3.2. Logistic Regression Analysis

Each language variable was compared for significance with the breastfeeding categories and adjusted for race and sex. Binary logistic regression and adjusted odds ratio was used for language variables which had binary outcomes (yes, no). The results are shown in Table 3. Ten language variables had binary outcomes. ‘No breastfeeding’ was used as reference. All variables in the exclusive breastfeeding category and the breastfed until 6 months but not exclusively had a significant *p*-value (all less than <0.001) with a positive adjusted odds ratio as compared to children who never breastfed. Children who were breastfed less than 6 months had significant difference in follows verbal direction (*p*-values of 0.004), follows two step commands (*p*-value of <0.001), understands ‘in’, ‘on’, and ‘under’ (*p*-values of 0.009), and points to things (<0.002). Children who were exclusively breastfed had 63% more odds to use two words together (*p*-value of <0.001) as compared to children who were never breastfed. Similarly, children who were breastfed until 6 months but not exclusively had 51.7% higher odds to use two words together as compared to children who were never breastfed (*p*-value of <0.001).

For all the rest of variables, children who were exclusively breastfed had 53.4% higher odds to use three words together, 87.2% to ask questions ‘who’, ‘what’, and ‘when’, 86.5% to ask questions ‘why’ and ‘how’, 70.6% to tell a story, 66.3% to understand ‘no’, 43.3% to follow verbal directions, 106% to point to things, 92.4% to follow two-step directions and 155% to understand ‘in’, ‘on’, and ‘under’; all with a significant *p*-value, as compared to children who were never breastfed.

Similarly, children who were breastfed until 6 months but not exclusively had higher odds of 186% to use three words together, 54% to ask questions ‘who’, ‘what’ and ‘when’, 51.5% to ask questions ‘why’ and ‘how’, 31.2% to tell a story, 190% to understand ‘no’, 118% to follow verbal directions, 79.2% to point to things, 93.8% to follow two-step directions, and 91.3% to understand ‘in’, ‘on’ and ‘under’; all with a significant *p*-value, as shown in Table 3, as compared to children who were never breastfed.

The highest percent odds increase is seen in the more complicated milestones, likely because these language milestones initially develop in this age group and indicate more advanced development.

Similar effect was seen in language milestones which had multiple possible outcomes. Multinomial regression was used for this analysis and the results are shown in Table 4. Reference categories were children who were never breastfed and children who have never done the intended milestone. Interestingly this significant association was not always seen in association with children who were exclusively breastfed until 6 months and more strongly seen in children who were breastfed until 6 months but not exclusively.

Compared to never breastfed children, children who were breastfed but less than 6 months had 138% higher odds of always naming emotions, 80.3% to always come up with words starting with the same sound, 85.2% to rhyme very well, and 54.1% to recognize the beginning of a sound, all with a *p*-value of <0.001.

Children who were breastfed until 6 months but not exclusively had 3.07 (confidence interval [C.I.]: 2.48–3.81) times higher odds of always naming emotions, 1.39 (C.I. of 1.22–1.60) times higher odds to always come up with words starting with the same sound, 1.92 (C.I. of 1.711–2.163) times higher odds to rhyme very well and 1.78 (C.I. of 1.52–2.10) times higher odds to recognize beginning of a sound as compared to children who were never breastfed, all with a *p*-value of <0.001. For this category, similar higher odds were seen in most of the times, half the time, and sometimes outcomes across the language variables-child can come up with words starting with the same sound, rhyme, express emotions, and recognize beginning of sound. Interestingly this positive odds ratio was not seen in the variable child can explain things clearly, in which children had lower odds to always, most of the times, half the time, and sometime explain things clearly. In this variable, positive association was seen in children who were exclusively breastfed.

## 4. Discussion

The current study is focused on exploring the impact of duration and exclusivity of breastfeeding on the development of language skills in 3–5-year-old children or preschoolers. Assessment of language outcomes in this age group is particularly important because many children prepare to enter the academic world during this age and an assessment of factors which can lead to better outcomes can help prepare them.

This study looked at a large number of children in a nationally representative sample in the United States. Studies in the past have explored the association of breastfeeding with various developmental sectors and our findings are consistent with previous studies that breastfeeding promotes language development [2,3,4]. However, the detailed break-down of individual milestones, differences seen amongst four different breastfeeding categories (never breastfed, breastfed less than 6 months, until 6 months and not exclusively and 6 months exclusively) and study of the 3-to-5 years age group exclusively has not been reported per our knowledge and literature search. The study also includes components of language which are important when evaluating for school readiness in early learning skills domain (child can recognize beginning of a sound, child can come up with words starting with the same sound, child can rhyme words) and social/developmental domain (child can recognize and name emotions, child can explain things clearly they have seen or done [9]). School readiness is an important concept to understand to meet child’s developmental needs at entry to kindergarten and promote healthy academic environment [14,15]. Understanding factors which can impact school readiness, like breastfeeding, is important for child’s long term academic success as well. The correlation of language variables associated with school readiness and breastfeeding has also not been done in previous studies.

The results of the study show that breastfeeding until 6 months, even if not exclusive has positive association with many language variables. The dataset is limited by how exclusive the duration of breastfeeding was in these 6 months, as well as when and what type of mixed feeding was introduced. However, a strong positive effect was seen consistently across many language variables across the two categories of breastfeeding (breastfed until 6 months but not exclusively, breastfed exclusively until 6 months). Positive effects were also seen in language milestones in children who were breastfed but not until 6 months, implying that breastfeeding even for a shorter duration has positive effect. The results of the analysis also indicate that the more complex a milestone is, the higher the adjusted odds ratio (AOR) is. This could be due to the age group of 3–5 years old, where these milestones are expected to develop, or due to complexity of the milestones, and more protective effects needed.

Choi et al. conducted studied association between breastfeeding and cognitive development in South Korea and found that exclusive breastfeeding followed by mixed feeding had higher odds of scoring better in developmental areas for communication (AOR: 4.12 [1.11–15.28]) and social interaction (AOR: 6.04 [1.05–34.66]) at 6 months as compared to infants who were never breastfed. Similar effects were found at 12 months. The study did not find significant difference in infants who were exclusively breastfed until 6 months [16].

When assessing language variables which are also components of school readiness, it is seen that compared to never breastfed, children who were breastfed until 6 months but not exclusively had more significant positive association. This effect was surprisingly not consistently in the exclusive breast-fed group. Almost all language variables had a positive association noted with breastfeeding except the variable-able to explain things clearly, which was noted to have a negative association with breastfeeding. More studies are needed to verify these associations, especially as the duration until which exclusive breastfeeding was done in this category is not known and what type of feeds were introduced. A similar effect was seen in the study mentioned above in different cognitive outcomes [16].

Our study had many strengths: studying the 3–5 years age group in a nationally representative U.S. sample, large sample size, analyzing language factors which affect school readiness, comparison across four breastfeeding categories, and analyzing individual milestones of language. Some of the limitations of the study include the retrospective nature of the study, which can introduce recall bias. The data collected do not include information on type of complementary feedings and when they were introduced, how long exclusive or partial breastfeeding was continued, or whether breastfeeding was continued beyond 6 months. Other potential influencers of language development, for example, genetics, NICU stay, parental education level, socioeconomic status, home language environment, access to day care, or gestation of the children, were not the part of the study design. These variables can influence the results and more studies are needed to analyze their impact on development of language milestones. The breastfeeding data were collected retrospectively from NSCH participants, hence, the possibility of recall bias.

Overall, the study shows that breastfeeding until 6 months is associated with higher positive odds ratio across multiple language variables in the preschool age group.

## 5. Conclusions

Adjusted weighted analysis shows positive association of breastfeeding with development of language variables in the 3–5 year age group. Exclusive breastfeeding until 6 months and breastfeeding until 6 months, even if not exclusive, has a positive association with development of language variables. Language variables associated with school readiness assessed in this age group also indicates a positive association with breastfeeding. This study can form the basis for prospective studies and studying the effect of other potential influencers like genetics, home language environment, etc., for analyzing the relationship between breastfeeding and language development in this age group.

## Figures and Tables

**Table 1:** Frequency and percentage of exposure variables.

Variables	Frequency	Percent
Age of Selected Child—In Years		
3 years	7489	33
4 years	7664	33.8
5 years	7512	33.1
Total	22,666	100
Sex of Selected Child		
Male	11,601	51.2
Female	11,065	48.8
Total	22,666	100
Race of Selected Child		
White alone	15,764	69.4
Black or African American alone	2923	12.9
American Indian or Alaska Native alone	249	1.1
Asian alone	1214	5.4
Native Hawaiian and Other Pacific Islander alone	180	0.8
Two or More Races	2336	10.3
Total	22,666	100
Breastfeeding for the first 6 months		
Never breastfed or given breast milk	4490	19.8
Breastfed less than 6 months	5209	23
Breastfed to six months, not exclusively	6425	28.3
Exclusively breastfed for first 6 months	6541	28.9
Total	22,666	100

**Table 2:** Weighted distribution of all variables across four breastfeeding categories.

	Category	Never Breastfed (*n*, %)	Breastfed < 6 mo (*n*, %)	Breastfed to 6 mo, Not Exclusive (*n*, %)	Exclusively Breastfed to 6 mo (*n*, %)
1	Age				
	Age 3	1314 (17.5%)	1774 (23.7%)	2189 (29.2%)	2213 (29.5%)
	Age 4	1521 (19.8%)	1817 (23.7%)	2199 (28.7%)	2127 (27.8%)
	Age 5	1654 (22.0%)	1619 (21.6%)	2037 (27.1%)	2202 (29.3%)
2	Sex				
	Male	2310 (19.9%)	2713 (23.4%)	3365 (29.0%)	3213 (27.7%)
	Female	2180 (19.7%)	2496 (22.6%)	3060 (27.7%)	3328 (30.1%)
3	Two words (language variable)				
	No	177 (25.0%)	229 (32.3%)	158 (22.3%)	144 (20.3%)
	Yes	4148 (19.4%)	4857 (22.7%)	6138 (28.6%)	6293 (29.4%)
4	Three words (language variable)				
	No	255 (27.2%)	292 (31.1%)	159 (17.0%)	232 (24.7%)
	Yes	4070 (19.2%)	4794 (22.6%)	6136 (28.9%)	6205 (29.3%)
5	Ask Questions: ‘Who’, ‘What’, ‘When’, ‘Where’ (language variable)				
	No	340 (26.9%)	376 (29.8%)	303 (24.0%)	244 (19.3%)
	Yes	3985 (19.1%)	4711 (22.6%)	5993 (28.7%)	6193 (29.7%)
6	Ask Questions: ‘Why’, ‘How’ (language variable)				
	Yes	3907 (19.0%)	4650 (22.6%)	5902 (28.7%)	6120 (29.7%)
	No	418 (26.7%)	437 (27.9%)	394 (25.2%)	317 (20.2%)
7	Tell a story (language variable)				
	Yes	3485 (18.7%)	4081 (21.9%)	5369 (28.8%)	5710 (30.6%)
	No	839 (24.0%)	1006 (28.8%)	927 (26.5%)	727 (20.8%)
8	Understand ‘no’ (language variable)				
	Yes	4180 (19.3%)	4929 (22.7%)	6231 (28.8%)	6327 (29.2%)
	No	145 (30.3%)	158 (33.1%)	65 (13.6%)	110 (23.0%)
9	Follow Verbal Directions (language variable)				
	Yes	4186 (19.3%)	4982 (23.0%)	6212 (28.6%)	6316 (29.1%)
	No	139 (31.0%)	105 (23.4%)	84 (18.7%)	121 (26.9%)
10	Point to Things (language variable)				
	Yes	4126 (19.2%)	4924 (22.9%)	6144 (28.6%)	6311 (29.3%)
	No	199 (31.1%)	162 (25.4%)	152 (23.8%)	126 (19.7%)
11	Follow Two Step Directions (language variable)				
	Yes	4118 (19.1%)	4943 (23.0%)	6150 (28.6%)	6293 (29.3%)
	No	206 (32.2%)	143 (22.4%)	146 (22.8%)	144 (22.5%)
12	Understand ‘In’, ‘On’, ‘Under’ (language variable)				
	Yes	4038 (19.0%)	4838 (22.8%)	6095 (28.7%)	6290 (29.6%)
	No	287 (32.5%)	248 (28.1%)	201 (22.8%)	147 (16.6%)
13	Rhyme Words (language variable)				
	Very well	837 (15.4%)	1066 (19.6%)	1716 (31.5%)	1823 (33.5%)
	Somewhat well	1689 (19.7%)	1957 (22.9%)	2442 (28.6%)	2465 (28.8%)
	This child cannot rhyme	1082 (22.7%)	1241 (26.1%)	1164 (24.5%)	1270 (26.7%)
	Not well	661 (20.4%)	771 (23.8%)	953 (29.5%)	848 (26.2%)
14	Child can come up with words starting with the same sound, age 3–5 years (language variable)				
	Always	1713 (19.4%)	1917 (21.7%)	2469 (27.9%)	2749 (31.1%)
	Most of the time	1117 (17.2%)	1445 (22.3%)	1958 (30.2%)	1968 (30.3%)
	About half the time	302 (18.1%)	402 (24.1%)	525 (31.5%)	438 (26.3%)
	Sometimes	646 (21.7%)	712 (24.0%)	799 (26.9%)	814 (27.4%)
	Never	503 (24.7%)	568 (27.9%)	521 (25.6%)	443 (21.8%)
15	Child can recognize beginning sound of a word, age 3–5 years (language variable)				
	Always	1823 (18.5%)	2182 (22.2%)	2779 (28.2%)	3057 (31.1%)
	Most of the time	1111 (17.2%)	1462 (22.7%)	2044 (31.7%)	1829 (28.4%)
	About half the time	348 (23.2%)	354 (23.6%)	372 (24.8%)	423 (28.3%)
	Sometimes	634 (22.9%)	646 (23.4%)	772 (27.9%)	711 (25.7%)
	Never	366 (24.8%)	404 (27.3%)	313 (21.2%)	395 (26.7%)
16	Child can recognize and name their own emotions, age 3–5 years (language variable)				
	Always	1668 (20.9%)	1899 (23.8%)	2125 (26.7%)	2274 (28.5%)
	Most of the time	1455 (16.5%)	1850 (20.9%)	2750 (31.1%)	2788 (31.5%)
	About half the time	389 (19.0%)	434 (21.3%)	603 (29.5%)	616 (30.2%)
	Sometimes	439 (20.6%)	525 (24.7%)	635 (29.8%)	530 (24.9%)
	Never	311 (33.7%)	307 (33.3%)	131 (14.2%)	173 (18.8%)
17	Child can explain things child has seen or done, age 3–5 years (language variable)				
	Always	2355 (18.9%)	2733 (22.0%)	3434 (27.6%)	3922 (31.5%)
	Most of the time	1144 (17.6%)	1380 (21.2%)	2112 (32.5%)	1861 (28.6%)
	About half the time	245 (22.9%)	261 (24.4%)	336 (31.4%)	228 (21.3%)
	Sometimes	259 (25.0%)	316 (30.5%)	246 (23.8%)	214 (20.7%)
	Never	287 (29.5%)	347 (35.7%)	157 (16.1%)	182 (18.7%)

**Table 3:** Weighted AOR for language variables having binary outcomes. CI: confidence interval. AOR: adjusted odds ratio.

Outcome Variable→	Breastfed < 6 Months		Breastfed Until 6 Months, Not Exclusively		Breastfed Exclusively Until 6 Months	
Language variables↓	(AOR [95% CI])	*p*-Value	(AOR [95% CI])	*p*-Value	(AOR [95% CI])	*p*-Value
Use Two Words Together	0.867 (0.71–1.06)	0.165	1.517 (1.22–1.89)	<0.001	1.631 (1.30–2.05)	<0.001
Use Three Words Together	1.004 (0.84–1.19)	0.964	2.286 (1.864–2.80)	<0.001	1.534 (1.28–1.85)	<0.001
Ask Questions: ‘Who’, ‘What’, ‘When’, ‘Where’	1.013 (0.87–1.18)	0.867	1.540 (1.31–1.81)	<0.001	1.872 (1.58–2.22)	<0.001
Ask Questions: ‘Why’, ‘How’	1.092 (0.95–1.26)	0.225	1.515 (1.31–1.75)	<0.001	1.865 (1.59–2.18)	<0.001
Tell a Story	0.930 (0.84–1.03)	0.171	1.312 (1.18–1.46)	<0.001	1.706 (1.53–1.9)	<0.001
Understand ‘No’	0.998 (0.79–1.26)	0.986	2.903 (2.16–3.91)	<0.001	1.663 (1.29–2.15)	<0.001
Follow Verbal Directions	1.470 (1.13–1.91)	0.004	2.181 (1.65–2.88)	<0.001	1.433 (1.11–1.84)	0.005
Point to Things	1.392 (1.12–1.72)	0.002	1.792 (1.44–2.23)	<0.001	2.066 (1.64–2.60)	<0.001
Follow Two-Step Directions	1.644 (1.32–2.05)	<0.001	1.938 (1.56–2.41)	<0.001	1.924 (1.55–2.39)	<0.001
Understand ‘In’, ‘On’, ‘Under’	1.266 (1.06–1.51)	0.009	1.913 (1.59–2.31)	<0.001	2.552 (2.08–3.14)	<0.001
Reference: Children not breastfed						

**Table 4:** Multinomial regression analysis between breastfeeding categories and language variables.

Breastfeeding Categories→	Breastfeeding <6 Months		Breastfeeding Until 6 Months But Not Exclusive		Exclusive Breastfeeding Until 6 Months	
Language Variables↓	AOR [95% CI]	*p*-Value	AOR [95% CI]	*p*-Value	AOR [95% CI]	*p*-Value
Child can come up with words starting with same sound, e.g., sock and sun						
Always	1.803 [1.56–2.08]	<0.001	1.396 [1.22–1.60]	<0.001	0.989 [0.862–1.13]	0.873
Most of the time	1.960 [1.69–2.27]	<0.001	1.690 [1.47–1.95]	<0.001	1.134 [0.98–1.31]	0.086
About half the time	1.614 [1.33–1.96]	<0.001	1.679 [1.39–2.03]	<0.001	1.167 [0.96–1.41]	0.115
Sometimes	1.415 [1.20–1.67]	<0.001	1.192 [1.02–1.40]	0.032	0.969 [0.83–1.14]	0.705
Rhyme words, e.g., cat, mat						
Not well	1.095 [0.96–1.25]	0.174	1.348 [1.185–1.534]	<0.001	1.023 [0.896–1.168]	0.737
Somewhat well	1.241 [1.12–1.38]	<0.001	1.353 [1.220–1.501]	<0.001	1.015 [0.914–1.127]	0.782
Very well	1.852 [1.65–2.08]	<0.001	1.924 [1.711–2.163]	<0.001	1.117 [0.989–1.263]	0.075
Child can recognize beginning sound of the word, e.g., ball starts with buh sound						
Always	1.541 [1.32–1.79]	<0.001	1.788 [1.52–2.10]	<0.001	1.086 [0.93–1.27]	0.296
Most of the time	1.503 [1.29–8.77]	<0.001	2.148 [1.82–2.54]	<0.001	1.185 [1.01–1.39]	0.04
About half the time	1.089 [0.89–1.33]	0.404	1.244 [1.01–1.54]	0.041	0.909 [0.74–1.12]	0.359
Sometimes	1.031 [0.86–1.23]	0.739	1.425 [1.18–1.71]	<0.001	0.922 [0.77–1.10]	0.373
Child can explain things clearly things they have seen or done						
Sometimes	0.757 (0.584–0.982)	0.036	0.771 (0.60–0.99)	0.041	1.321 (0.99–1.75)	0.053
About half the time	0.697 (0.54–0.90)	0.006	0.607 (0.47–0.78)	<0.001	1.764 (1.34–2.33)	<0.001
Most of the time	0.406 (0.33–0.49)	<0.001	0.397 (0.33–0.48)	<0.001	1.387 (1.11–1.73)	0.004
Always	0.393 (0.32–0.48)	<0.001	0.372 (0.31–0.45)	<0.001	1.064 (0.85–1.33)	0.580
Child can recognize and name emotions						
Sometimes	2.113 (1.69–2.65)	<0.001	3.463 (2.73–4.39)	<0.001	1.199 (0.98–1.47)	0.079
About half the time	2.704 (2.16–3.39)	<0.001	3.703 (2.91–4.72)	<0.001	1.102 (0.89–1.36)	0.363
Most of the time	3.288 (2.69–4.00)	<0.001	4.531 (3.66–5.61)	<0.001	1.263 (1.06–1.50)	0.008
Always	2.388 (1.96–2.91)	<0.001	3.071 (2.48–3.81)	<0.001	1.148 (0.97–1.36)	0.114
Reference categories: For breastfeeding categories: never breastfed. For language variable: Never achieved the milestone.						

AOR: adjusted odds ratio. CI: confidence interval.

## Data Availability

The data are publicly available at the NSCH website and this research utilized the 2022–2023 NSCH dataset. It can be requested from the official website.

## Abbreviations

The following abbreviations are used in this manuscript:

U.S.	United States
A.O.R	adjusted odds ratio
C.I.	confidence interval
NSCH	National Survey of Child Health
Mo	months
